# Predicting high health care use in patients with spinal disorder in secondary care: model development and validation

**DOI:** 10.1097/PR9.0000000000001382

**Published:** 2026-01-16

**Authors:** Lise Grethe Kjønø, Karin Magnusson, Marianne Bakke Johnsen, Kjersti Storheim, Stine Haugaard Clausen, Maja Wilhelmsen, Søren O'Neill, Are Hugo Pripp, Bjørnar Berg, Christian Volmar Skovsgaard, Jan Hartvigsen, Margreth Grotle, Ørjan Nesse Vigdal

**Affiliations:** a Department of Rehabilitation Science and Health Technology, Faculty of Health Sciences, Oslo Metropolitan University, Oslo, Norway; b Cluster for Health Services Research, Norwegian Institute of Public Health, Oslo, Norway; c Clinical Epidemiology Unit, Department of Clinical Sciences, Orthopaedics, Faculty of Medicine, Lund University, Lund, Sweden; d Department of Physical Medicine and Rehabilitation, Oslo University Hospital, Oslo, Norway; e Division of Clinical Neuroscience, Department of Research and Innovation, Oslo University Hospital, Oslo, Norway; f Center for Muscle and Joint Health, Department of Sports Science and Clinical Biomechanics, University of Southern Denmark, Odense, Denmark; g Faculty of Health Sciences, Department of Clinical Medicine, UiT—The Arctic University of Norway, Tromsø, Norway; h Department of Rehabilitation, University Hospital of North Norway, Tromsø, Norway; i Medical Spinal Research Unit, Spine Centre of Southern Denmark, Lillebaelt Hospital - University Hospital of Southern Denmark, Kolding, Denmark; j Faculty of Health Sciences, Oslo Metropolitan University, Oslo, Norway; k Oslo Centre of Biostatistics and Epidemiology, Research Support Services, Oslo University Hospital, Oslo, Norway; l Department of Public Health, Danish Centre for Health Economics, University of Southern Denmark, Odense, Denmark; m Chiropractic Knowledge Hub, Odense, Denmark; n Department of Regional Health Research, University of Southern Denmark, Odense, Denmark

**Keywords:** Spinal disorders, Registry data, Epidemiology, High health care utilization, Prediction model, Validation

## Abstract

**Introduction:**

Spinal disorders are among the biggest contributors to health care utilization (HCU).

**Objective:**

To develop and externally validate a prediction model for high all-cause HCU (75th percentile during 1 year after the index date) among patients with spinal disorders visiting multidisciplinary secondary care clinics.

**Methods:**

We developed and internally validated the model using the Norwegian Neck and Back Registry, including patients registered between January 1, 2016, and December 31, 2020, linked with national health registries (N = 9092). For external validation, we used data from the Danish SpineData Registry, linked with national registries, for the same period (N = 34,853). We assessed Nagelkerke *R*^*2*^, discrimination (area under receiver operating characteristics curve [AUC]), and calibration (calibration-in-the-large [CITL], slope, and calibration plot).

**Results:**

The final model included sex, nationality, education, physical activity, smoking, prior HCU, work status, disability, health-related quality of life, medicine use, diagnosis, kinesiophobia, and comorbidity. It demonstrated acceptable discrimination (AUC 0.78, 95% confidence interval [CI], 0.77–0.78), an *R*^2^ of 0.26, and good calibration after internal validation. Upon external validation, the model demonstrated excellent discrimination (AUC 0.81, 95% CI 0.80–0.81) and an *R*^2^ of 0.31. The calibration slope was 1.08 (95% CI 1.06–1.11) and CITL was 0.16 (95% CI 0.12–0.19). Predicted probabilities closely matched observed probabilities across all deciles in internal validation, with slight underestimation of high HCU in the top 3 deciles during external validation.

**Conclusion:**

Overall, the model shows promise in predicting high HCU in patients with spinal disorders referred to secondary care but requires further testing and validation in implementation settings before recommendation.

## Abstract 2

Supplemental Digital Content is Available in the Text.

A clinical prediction model developed to predict high health care utilization among people with spinal disorders in secondary care performed well upon internal and external validation.

## 1. Introduction

Spinal disorders remain a major contributor to disability and health care utilization (HCU) globally.^23^ Among patients with spinal disorders in Norway and Denmark, a minority of patients account for most of the total HCU.^36,47^ Despite the direct cost of HCU for spinal disorders reaching over 600 million EUR in Norway in 2019,^37^ care-seeking behavior is not well understood.

Factors such as higher pain, disability, comorbidity, work status, and smoking are associated with higher HCU in primary care populations and in the general population.^9,11,35,42,70^ However, we have limited evidence from the secondary care setting, which is more resource-intensive and expensive than primary care. We recently reported that a substantial proportion of patients with spinal disorders referred to a specialist evaluation in secondary care continue to use health care services over the following 2 years, particularly physical therapy consultations.^40^ In addition, another study of this patient population found that up to 80% reported nonrecovery 6 months after the evaluation, indicating the need for effective long-term management strategies.^39^ Patients referred to secondary care are likely to have more pain and disability, longer pain duration, and higher proportion of neurological symptoms than patients treated in primary care^27,66^ all of which can contribute to increased HCU. Further, studies have shown that musculoskeletal pain often coexists with other chronic conditions, leading to comorbidity, increased complexity of treatment, and higher HCU.^48^

Understanding predictors of future HCU and reliably predicting levels of HCU is important for health care service planners when assigning funding and planning care pathways. This is especially important in the context of an aging population and increasing economic constraints in health care systems. Moreover, reliable predictions of HCU derived from a prediction model can be a tool for health care providers to discuss health care seeking with patients.

The aim of this study was to develop and internally and externally validate a prediction model for high all-cause health care utilization among patients with spinal disorders visiting secondary care.

## 2. Methods

### 2.1. Study design

The study is a prospective, multicenter, registry-based prognostic study on adults with spinal disorders visiting specialized, multidisciplinary outpatient clinics in Norwegian and Danish secondary care. The study was developed and designed following the PROGnosis RESearch Strategy (PROGRESS) framework for developing and validating prognostic models and reported in accordance with the Transparent Reporting of a Multivariable Prediction Model for Individual Prognosis or Diagnosis (TRIPOD + AI) statement.^14,62^ The study is part of the AID-Spine project, which was approved by the ethics committee of the Health Region of South-East Norway (2022/371282) and by the Danish Data Protection Agency (notification number 11.856) and by the region of Southern Denmark (Journal nr. 23/38730). All participants provided written consent for participation in the clinical registries and for data linkage.

### 2.2. Data sources

#### 2.2.1. Model development and internal validation

Predictors in the development and internal validation cohort were collected from the Norwegian Neck and Back Registry (NNRR) between January 1, 2016 and December 31, 2020. The NNRR is a national medical quality registry including patients who are conservatively treated at the multidisciplinary physical medicine outpatient clinics in Norwegian hospitals.^1,65,66^ The NNRR includes both patient self-report data and data provided by the health care practitioner. If a patient had more than 1 entry in the NNRR, we handled the first consultation as the index consultation.

Data on HCU were collected from 2 public registries: The Norwegian Control and Payment of Health Reimbursements Database (KUHR) and The Norwegian Patient Registry (NPR). The KUHR registry registers all HCU in all publicly funded primary care.^63^ In the current study, we limited HCU in primary care to primary care physicians (including general practitioners and municipal urgent care visits), physical therapists (including manual therapists and psychomotor physical therapists), and chiropractors. The NPR is a health register that covers all inpatient, day patient, and outpatient specialist health services in Norway.^64^ We included inpatient and outpatient HCU, including services provided by self-employed specialists with reimbursement contracts. Health care utilization was collected for the 12 months preceding the index consultation registered in NNRR, and 12 months after the index consultation. Data on deaths and individuals who emigrated were obtained from Statistics Norway (SSB), which provides status as of January 1st each year. Linkage between NNRR, KUHR, NPR, and SSB was done using Norwegian national identity numbers.

#### 2.2.2. External validation

For external validation, we used data from the SpineData registry between January 1, 2016 and December 31, 2020 and Danish national health registry data. SpineData is a Danish clinical registry that systematically collects data on individuals with spinal pain as part of routine clinical practice at the Spine Centre of Southern Denmark. This outpatient spine clinic, operating within the secondary health care system, serves approximately 1.2 million residents in the Region of Southern Denmark.^33^ The clinic conducts comprehensive multidisciplinary assessments of patients with severe or long-lasting spinal pain referred by general practitioners, chiropractors, and medical specialists in primary care.

We linked patient-reported data on work, lifestyle, disability, and pain from the SpineData questionnaire^33^ with individual-level data from national registries using the Danish national civil registration number.^58^ Patient-reported data, as well as register data were collected, and variables were defined to make the Danish and Norwegian data as comparable as possible. We obtained data on general practice, physical therapy, and chiropractor consultations from the Danish National Health Service Registry,^56^ in- and outpatient consultations, and surgical history from the Danish National Patient Registry,^59^ prescribed medication from the National Prescription Registry,^34^ marital status from the Population Register,^58^ highest achieved level of education from the Population Education Registry,^32^ annual disposable personal income, and working status from the Income Registry,^4^ and the Danish Registry for Evaluation of Marginalization.^29^

### 2.3. Study population

Adults aged 18 to 70 years, referred to specialist secondary care clinics for back and/or neck pain, with or without radiating pain, were included. The age cutoff of 70 years was applied, as HCU for individuals above this age is often missing in the KUHR database due to nursing home residency. Individuals who had died or emigrated by January 1st of the year after the index consultation, or failed to complete the baseline questionnaire, were excluded.

### 2.4. Outcome

The outcome to be predicted was high HCU during the first 12 months after the index consultation. To strike a balance between ensuring the identification of high health care users and maintaining enough observations for robust analysis, we defined high HCU as the highest quartile (75th percentile) of HCU. This definition aligns with other studies assessing HCU^11^ and the associated costs.^5,18,35,38^ We chose 12 months after because a previous study within our group showed that HCU is most prevalent in the 12 months after the secondary care consultation.^40^

Health care utilization was defined as all-cause face-to-face or video consultations with health care providers in primary and secondary care. If patients had more than 1 registration per health care provider for 1 day, this was counted as 1 consultation. We counted registrations with different health care providers in primary care on the same day as different consultations. We excluded registrations of phone contacts, prescriptions, and administrative tasks. The number of consultations with different health care providers was summed to a total count, which was then dichotomized at the 75th percentile.

### 2.5. Candidate predictors

Candidate predictors were selected from available variables in both the NNRR and Danish registries, based on evidence and author consensus. For predictors with limited evidence, discussions among the authors, including medical doctors, physical therapists, and chiropractors, evaluated their clinical relevance. Predictors were assessed at baseline, while the outcome was independently obtained from health registries, ensuring blinding of predictor assessment to outcome status. We included 22 candidate predictors, categorized by Andersen's Behavioral Model of Health Services Use,^3^ which suggests that 3 main domains influence HCU:

(1) Individual predisposing predictors: Age,^10,42,47,70,74^ sex,^10,22,47,70^ country of origin,^42,70^ marital status,^47^ education,^42,47,70^ physical activity levels,^11,54^ smoking status,^11,70,74^ and HCU previous year.^10,11,47^
(2) Individual enabling predictors: Work status,^9,11,42,47^ applied for disability pension or pending insurance claim issue,^30^ previous sick leave for similar complaint,^9,42^ level of work satisfaction,^51^ and level of physically heavy work.^2^
(3) Individual needs predictors: Pain duration,^42^ pain intensity,^9,22,35,47^ disability by Oswestry Disability Index^21^ and Neck Disability Index,^9,22,35,41,43,47,68^ previous spinal surgery,^46^ prescription of pain medication,^42^ pain location,^10,42,70^ kinesiophobia by Fear Avoidance Beliefs Questionnaire—Physical activity subscale,^69,71^ health-related quality of life by EQ5D,^20,35,42,47,74^ and comorbidities.^11,35,42,47,50,70,74^

Predictor definitions, measurement levels, and parameters are detailed in Supplementary Table S1, http://links.lww.com/PR9/A367. Comorbidity definitions are presented in Supplementary Table S2, http://links.lww.com/PR9/A367.

### 2.6. Sample size consideration

#### 2.6.1. Development cohort

The sample size was restricted to available NNRR data (n ≈ 9000). An a priori sample size calculation was performed to evaluate the adequacy of the dataset and guide how many predictors could be included. We used the *pmsampsize* package for R,^19^ and assumed an area under receiver operating characteristics curve (AUC) of 0.70, 48 predictor parameters, and an outcome prevalence of 25%. The calculation assumes a shrinkage of predictor effects of 10% or less, and an absolute difference of 0.05 in Nagelkerke *R*^*2*^ between the apparent and adjusted model.^19^ The calculation indicated a minimal sample size of n = 4391.

#### 2.6.2. External validation cohort

The sample size in the external validation cohort was restricted to available SpineData (n ≈ 35,000). We assessed the adequacy of the sample size using input values from the prediction model after internal validation, using the *pmvalsampsize* package for R.^19^ The mean (standard deviation) of the linear prediction after internal validation was −1.4 (1.1). We assumed an AUC of 0.7, and a calibration slope of 0.9. This yielded a minimum sample size of n = 3085.

### 2.7. Statistical analyses

Missing predictor data were handled using multiple imputations by chained equations and predictive mean matching in 44 datasets for both the development and external validation samples. Missing data were assumed to be missing at random (MAR), and auxiliary variables, including the outcome variable, postal code, and regional health trust, were included to strengthen the MAR assumption and improve the imputation models.^67^ Number of imputations was based on percentage of participants with missing variables.^52^ Convergence was assessed with convergence plots, and distribution of imputed vs observed values was checked (Supplementary Figs. S1–S2, http://links.lww.com/PR9/A367).^67^

Descriptive baseline data are reported as mean and standard deviation or median and interquartile range for continuous variables, depending on distribution, and frequencies and proportions for categorical variables. Descriptive HCU data throughout follow-up is reported as mean and standard deviation, as well as median and range. The Lorenz curve visualizes HCU distribution.^44,45^ We assessed sample relatedness by comparing distributions of predictors, outcome, and the mean and standard deviation of the linear predictors in both cohorts.^15^ Distribution of probabilities among patients with and without high HCU was presented with density plots.

#### 2.7.1. Model development and internal validation

We developed a multivariable logistic regression model using a backward selection approach. All candidate predictors were entered into the model simultaneously and removed based on a moderately strict *P*-value of <0.157 (Akaike Information Criterion).^12,55,57^ This procedure was performed separately in all imputed datasets. Model specification and performance measures were pooled using the median of the *P*-values (MPR) method, since we included categorical predictors with more than 2 levels.^49^ We evaluated the model's pooled performance measures of discrimination (AUC) and overall performance (Nagelkerke *R*^*2*^). Area under receiver operating characteristics curve values of 0.7 to 0.8 were considered acceptable, and 0.8 to 0.9 were considered excellent.^31^ Nonlinearity was assessed by applying restricted cubic splines with 3 and 5 knots to continuous candidate predictors.

Internal validation followed Collins et al.^13^ procedure to correct for overoptimism. We created 500 bootstrap samples from the original sample. Within each bootstrap sample, multiple imputation was performed as described above, the model development (including backward selection process across the imputed datasets) was performed, and the performance measures were pooled across the imputed datasets. The model developed in each bootstrap sample was then tested in the original datasets. We then calculated the average difference (optimism) in performance produced in the bootstrap and original datasets to derive optimism-adjusted performance measures. The estimated slope value from the bootstrapping served as a uniform shrinkage factor multiplied with the prediction model coefficients to arrive at an optimism-adjusted model. This optimism-adjusted model was then applied in the original imputed datasets to provide a calibration plot for the internally validated prediction model.^13^

#### 2.7.2. External validation

The optimism-corrected model from the internal validation was assessed in the external validation cohort by applying the exact predictor coefficients from the internally validated model to the external validation cohort, thus calculating the linear predictor, and assessing its performance (discrimination, overall performance, and calibration).^53^

#### 2.7.3. Additional analyses

Due to the unavailability of previous HCU data for clinicians at the point of care (as these data come from registry sources rather than the NNRR), we developed 2 models excluding this predictor: 1 model by excluding previous HCU from the original model, and another without this predictor from the start. For internal validation, we employed 100 bootstrap samples to enhance computational efficiency. Performance estimation methods were conducted as previously described in the original model and were applied consistently across both internal and external validation. To assess the robustness of the main analyses, we conducted sensitivity analyses using the 90th percentile as a cutoff to define high HCU and performed complete case analyses (CCA).

## 3. Results

**Figure 1.**
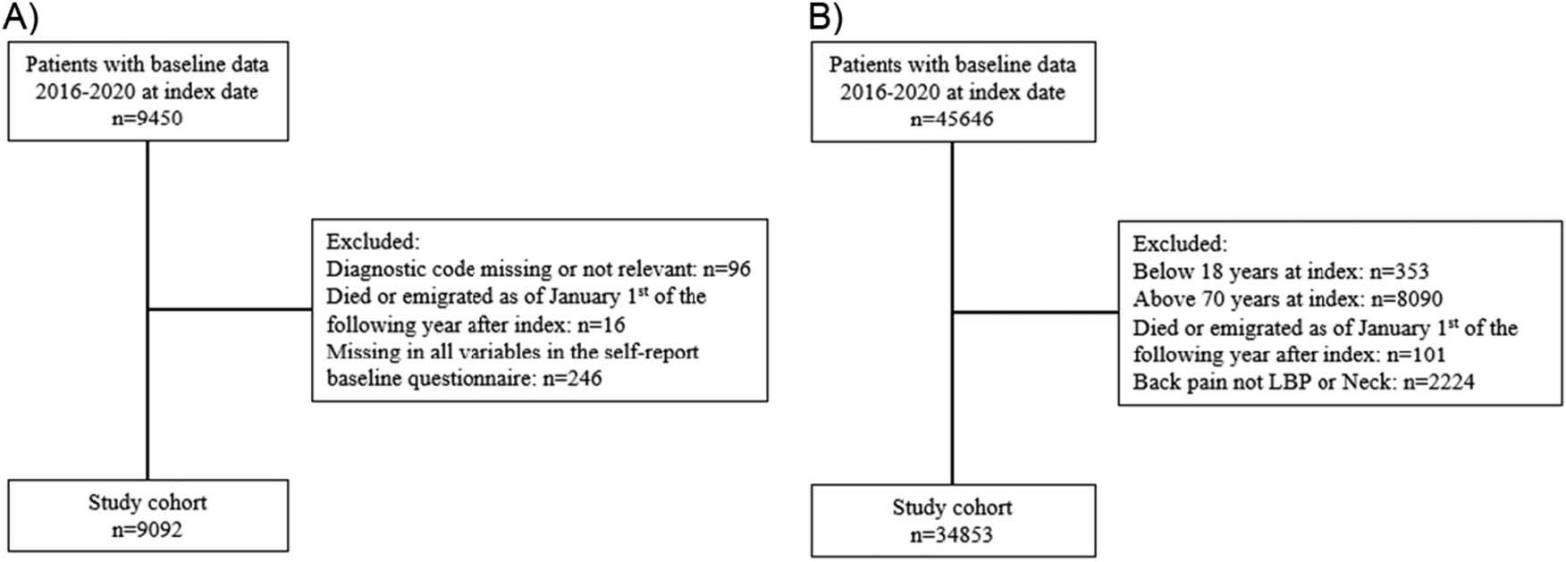
Flowchart of participants included in the (A) development sample and (B) external validation sample.

**Figure 2.**
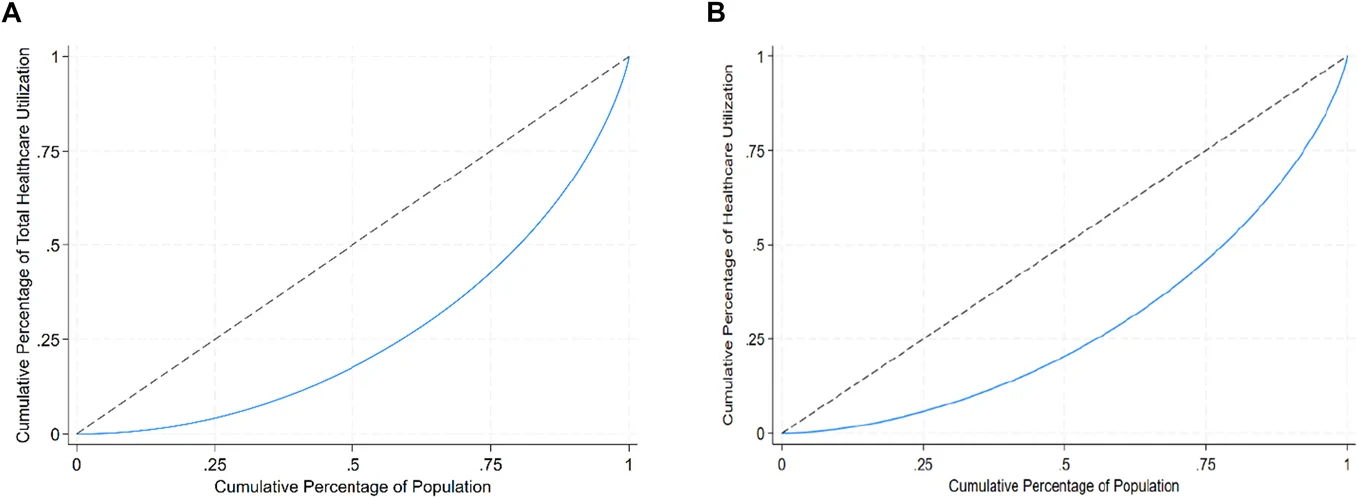
Lorenz curve of the distribution of HCU in (A) the internal validation sample and (B) the external validation sample. The *x*-axis represents the cumulative proportion of patients, ranked from the lowest to the highest levels of HCU, whereas the *y*-axis represents the cumulative proportion of total HCU. Blue line: Lorentz curve; dashed line: line of equality. HCU, health care utilization.

Of the 9450 registered patients in NNRR between 2016 and 2020, we included 9092 in the prediction model development and internal validation (Fig. 1A). The external validation cohort included 34 853 patients (Fig. 1B). Baseline characteristics, including distribution of candidate predictors, from both samples, are summarized in Table 1. In general, there were minor differences between the Norwegian and Danish spine samples. However, patients in the development sample were younger, more educated, and more likely to be on sick leave. They had longer pain durations but with lower intensity, they used more prescribed medications, and fewer patients had comorbidities. Descriptives of HCU before and after index consultation are presented in Supplementary Table S3, http://links.lww.com/PR9/A367. The cumulative distribution of HCU in the 2 cohorts is presented in Figure 2, which shows that the top 25% users in the sample were responsible for almost 60% of the total HCU throughout the follow-up year.

**Table 1:** Baseline characteristics of all participants in the development and validation cohorts.

	Development cohort (n = 9092)		Validation cohort (n = 34,853)	
	All participants	Missing, n (%)	All participants	Missing, n (%)
Predisposing factors				
Age, mean (SD)	43.8 (12.6)	0 (0)	49.5 (12.9)	0 (0)
Sex female, n (%)	5219 (57.4)	0 (0)	19,281 (55.1)	0 (0)
Country of origin, n (%)		707 (7.8)		0 (0)
Native	7672 (84.4)		30,840 (88.5)	
Other	713 (7.8)		4013 (11.8)	
Marital status, n (%)		103 (1.1)		0 (0)
Married/cohabiting	6449 (70.9)		23,582 (67.7)	
Single	2540 (27.9)		11,271 (32.3)	
Education level, n (%)		83 (0.9)		669 (1.9)
Primary school	1115 (12.3)		9068 (26.0)	
High school	4404 (48.4)		16,537 (47.4)	
Higher education	3490 (38.4)		8579 (24.6)	
Physical activity, n (%)		325 (3.6)		2560 (7.3)
Sedentary	1113 (12.2)		7329 (21.0)	
Light, for at least 4 h per week	5680 (62.5)		18,084 (51.9)	
Moderate, for at least 4 h per week	1537 (16.9)		6378 (18.3)	
Hard, competitive several times per week	436 (4.8)		502 (1.4)	
Smoking daily, yes, n (%)	1508 (16.6)	202 (2.2)	8324 (23.9)	2037 (5.8)
HCU previous year		0 (0)		0 (0)
Q1 (∼25%)	0–6 visits		0–11 visits	
Q2 (∼25%)	7–13 visits		12–19 visits	
Q3 (∼25%)	14–23 visits		20–29 visits	
Q4 (∼25%)	24–200 visits		30–350 visits	
Enabling factors				
Work status, n (%)		246 (2.7)		1996 (5.7)
Working or student	3625 (39.8)		14,388 (41.3)	
Stay at home or retired	335 (3.7)		6845 (19.6)	
Sick leave or unemployed	3497 (38.5)		6856 (19.7)	
Work assessment allowance or disability pension	1389 (15.3)		4768 (13.7)	
Applied for disability pension or insurance claim issue, n (%)	680 (7.5)	1063 (11.7)	1673 (12.9)	21,929 (62.9)
Previous sick leave, n (%)		1480 (16.3)		10,995 (31.5)
No	3993 (43.9)		13,164 (37.8)	
Yes	3619 (39.8)		10,694 (30.7)	
Satisfied with work (0–10), mean (SD)	7.6 (2.4)	953 (10.5)	7.7 (2.5)	2543 (17.7)
Physically heavy work (0–10), mean (SD)	4.6 (3.3)	929 (10.2)	4.8 (3.0)	18,306 (52.5)
Need factors				
Pain duration, n (%)		117 (1.3)		1768 (5.1)
<3 mo	445 (4.9)		6152 (17.7)	
3–11 mo	2764 (30.4)		12,373 (35.5)	
≥12 mo	5766 (63.4)		14,560 (41.8)	
Pain intensity (NRS 0–10), mean (SD)	5.3 (2.3)	206 (2.3)	6.7 (2.1)	1009 (2.9)
Disability (ODI/NDI 0–100), mean (SD)	34.6 (15.2)	283 (3.1)	36.1 (17.3)	13,387 (38.4)
Previous surgery in neck, back, or pelvis, n (%)		296 (3.3)		0 (0)
Yes	1013 (11.1)		3971 (11.4)	
No	7783 (85.6)		30,882 (88.6)	
Prescription pain medication, yes, n (%)	4707 (51.8)	213 (2.3)	13,555 (38.9)	0 (0)
Pain location, n (%)		0 (0)		0 (0)
Back pain	5701 (62.7)		26,328 (75.5)	
Neck pain	2431 (26.7)		8525 (24.5)	
Back and neck pain	960 (10.6)		0 (0)*	
Kinesiophobia (0–10), mean (SD)	5.3 (3.4)	893 (9.8)	4.9 (3.4)	2483 (7.1)
HRQoL (EQ5D −0.594 to 1), median (IQR)	0.656 (0.159–0.760)	694 (7.6)	0.660 (0.389–0.771)	2898 (8.3)
Comorbidity, n (%)		0 (0)		0 (0)
0	3468 (38.1)		12,674 (36.4)	
1	3690 (40.6)		8905 (25.6)	
2	1446 (15.9)		5737 (16.5)	
3	399 (4.4)		3506 (10.1)	
4–6	89 (1.0)		4031 (11.6)	

* In the Danish data, patients completed either a low back or a neck pain questionnaire, which defined their pain location as either low back or neck/thoracic pain, without the option to indicate both.

EQ 5D, EuroQol-5 dimension; HCU, health care utilization; HRQoL, Health-related quality of life; IQR, interquartile range; NDI, neck disability index; NRS, numeric rating scale; ODI, Oswestry disability index; SD, standard deviation.

### 3.1. Model development and internal validation

The final prediction model is detailed in Table 2. The predictors remaining in the prediction model were sex, country of origin, education level, physical activity level, smoking, HCU previous year, work status, disability, prescription medicine use, pain location, kinesiophobia, health-related quality of life, and comorbidity. Applying restricted cubic splines did not improve model performance; thus, we treated all continuous predictors linearly. When assessing the strength of association, the most important predictor was HCU during the previous year. Box 1 gives a demonstration of how the model can be used to calculate predictions for high HCU within 1 year for individual patients.

**Table 2:** Final multivariate logistic regression model for predicting high health care utilization during 1 year after index specialist care consultation.

Term	Odds ratio (95% CI)	*P*	Coefficients (SE)	OA coefficients
Intercept	0.04 (0.02, 0.05)	0.00	−3.34 (0.21)	−3.29
Predisposing factors				
Sex female (ref: male)	1.61 (1.43, 1.81)	0.00	0.48 (0.06)	0.47
Non-native origin (ref: native)	0.82 (0.67, 1.02)	0.07	−0.19 (0.11)	−0.19
Education level (ref: primary school)				
High school	1.12 (0.93, 1.33)	0.23	0.11 (0.09)	0.11
Higher education	1.36 (1.13, 1.63)	0.00	0.31 (0.09)	0.30
Physical activity level (ref: hard)				
Moderate	0.97 (0.72, 1.31)	0.84	−0.03 (0.15)	−0.03
Light	1.08 (0.82, 1.43)	0.57	0.08 (0.14)	0.08
Sedentary	1.35 (0.99, 1.84)	0.06	0.30 (0.16)	0.29
Smoking (ref: nonsmoking)	0.87 (0.75, 1.01)	0.06	−0.14 (0.08)	−0.14
Enabling factors				
HCU previous year (ref: Q1)				
Q2	1.88 (1.54, 2.30)	0.00	0.63 (0.10)	0.62
Q3	3.26 (2.69, 3.97)	0.00	1.18 (0.10)	1.16
Q4	10.65 (8.82, 12.86)	0.00	2.37 (0.10)	2.33
Work status (ref: working/student)				
Stay at home/retired	1.59 (1.20, 2.12)	0.00	0.47 (0.15)	0.46
Sick leave/unemployed	1.59 (1.39, 1.82)	0.00	0.46 (0.07)	0.46
Work assessment allowance/disability pension	1.33 (1.12, 1.58)	0.00	0.28 (0.28)	0.28
Need factors				
Disability (ODI/NDI 0–100)	1.01 (1.00, 1.01)	0.00	0.01 (0.00)	0.01
Prescription of pain medication (ref: not taking prescription pain medication)	1.11 (0.99, 1.25)	0.07	0.11 (0.06)	0.10
Pain location (ref: back pain)				
Neck pain	1.26 (1.11, 1.44)	0.00	0.23 (0.06)	0.23
Back and neck pain	1.29 (1.08, 1.53)	0.00	0.25 (0.09)	0.25
Kinesiophobia (0–10)	0.98 (0.97, 1.00)	0.05	−0.02 (0.01)	−0.02
HRQoL (EQ5D −0.594 to 1)	0.61 (0.49, 0.77)	0.00	−0.49 (0.11)	−0.48
Comorbidity (0–6)	1.10 (1.04, 1.17)	0.00	0.10 (0.03)	0.09

Optimism-adjusted (OA) coefficients are adjusted after internal validation through 500 bootstrap samples.

EQ 5D, EuroQol-5 dimension; HCU, health care utilization; HRQoL, health-related quality of life; NDI, neck disability index; ODI, Oswestry disability index.

Box 1. **Demonstration of prediction calculation.**

**Demonstration of equations for predicting high HCU within 12 months in individual patients with spinal disorders**

High HCU=−3.29+sex (0.47 if female) +country of origin (−0.19 if non−native)+education level (0.11 if high school,0.30 if higher education)+physical activity level (−0.03 if moderate,0.08 if light,0.29 if sedentary) +smoking (−0.14 if smoker)+HCU previous year (0.62 if Q2,1.16 if Q3,2.33 if Q4)+work status (0.46 if stay at home,0.46 if sick leave,0.28 if work assessment allowance)+0.01 ×(disability)+−0.48×(HRQoL)+prescription medicine use (0.10 if yes)+pain location (0.23 if neck pain,0.25 if back & neck pain)+−0.02×(kinesiophobia)+0.09 ×(comorbidity)

**Example 1**

Patient 1 is a Norwegian woman with back pain. She has primary school education, is currently on sick leave, lives a sedentary lifestyle, smokes, and had 24 health care visits in the previous year. She has a disability score of 56, HRQoL score of 0.10, kinesiophobia score of 7, is using prescribed medication, and has 2 comorbidities

High HCU would be estimated as follows:

High HCU=−3.29+(0.47×1) (female)+(−0.19×0) (native)+0 (primary school education)+0.29 (sedentary lifestyle)
+(−0.14×1) (smoker)+2.33 (HCU previous year)+0.46 (sick leave)+(0.01×56) (disability)+(−0.48×0.10) (HRQoL)
+(0.10×1)(prescription medicine use)+0 (back pain)+(−0.02×7) (kinesiophobia)+(0.09×2) (comorbidities)=0.772
  $$\text{Probability}\;\text{of}\;\text{high}\;\text{HCU}=\frac{\exp\left(0.772\right)}{1+\exp\left(0.772\right)}=0.684$$

Thus, patient 1 has a 68.4% probability of high HCU in the next 12 months

**Example 2**

Patient 2 is a Norwegian woman with back pain. She has higher education, is currently working, engages in hard physical activity, is a nonsmoker, and had 5 health care visits in the previous year. She has a disability score of 35, HRQoL score of 0.65, kinesiophobia score of 5, is not using prescribed medication, and has no comorbidities

High HCU would be estimated as follows:

High HCU=−3.29+(0.47×1) (female)+(−0.19×0) (native)+0.30 (higher education)
+0 (hard physical activity)+(−0.14×0) (non−smoker)+0 (HCU previous year)+0 (working)
+(0.01×35) (disability)+(−0.48×0.65) (HRQoL)+(0.10×0) (prescription medicine use)
+0 (back pain)+(−0.02×5) (kinesiophobia)+(0.09×0) (comorbidities)=−2.582
  $$\text{Probability}\;\text{of}\;\text{high}\;\text{HCU}=\frac{\exp\left(-2.582\right)}{1+\exp\left(-2.582\right)}=0.070$$

Thus, patient 2 has a 7.0% probability of high HCU in the next 12 months

HCU, health care utilization; HRQoL, health-related quality of life.

### 3.2. Internal and external validation

**Figure 3.**
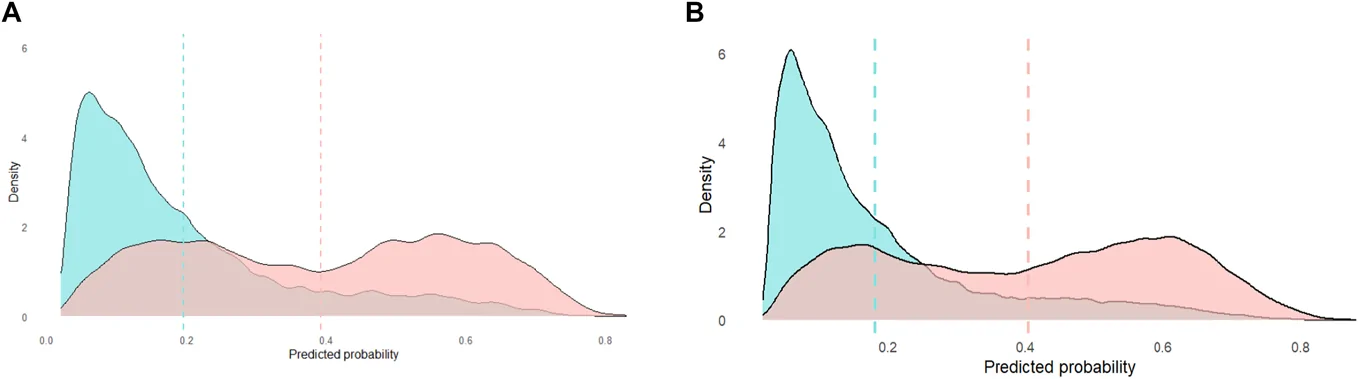
Distribution of predicted probabilities for high HCU among patients with (blue) and without (red) high HCU in (A) the internal validation sample and (B) the external validation sample. The *y*-axis shows estimated probability density, which represents the relative concentration of patients along the predicted probability scale for high HCU. Higher values on the *x*-axis reflect a greater model-estimated risk of high HCU. The darker shaded area represents where the 2 distributions overlap. Vertical dashed lines show the mean predicted probability in each group. HCU, health care utilization.

**Figure 4.**
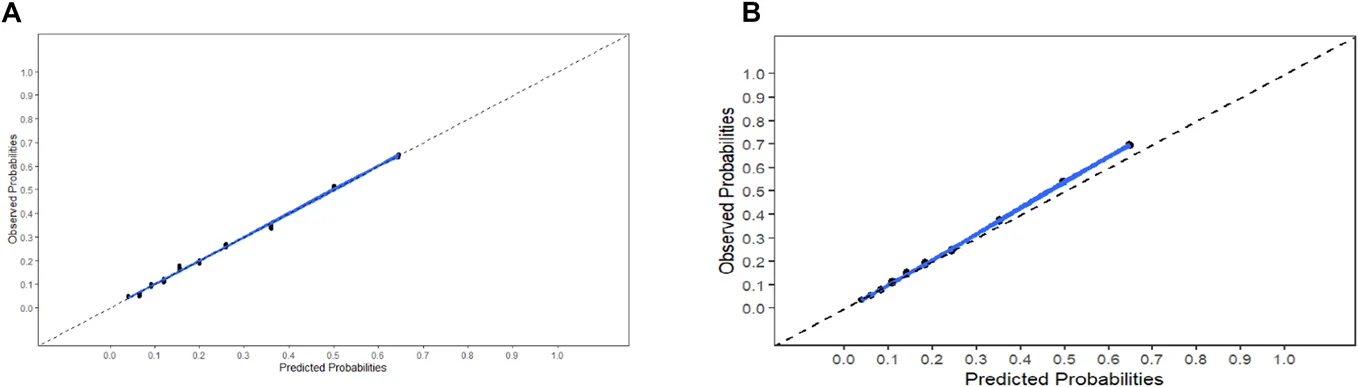
Calibration plots for the prediction model for high HCU in (A) the internal validation sample and (B) the external validation sample. Dots represent the agreement between observed and predicted high HCU. The 45° line represents perfect predictions. Dots represent deciles of predicted probabilities. The blue solid line is a smoothed calibration line, showing the average agreement between the observed and predicted number of patients with high HCU. HCU, health care utilization.

Figure 3 illustrates the distribution of predicted probabilities, showing that patients with high HCU in the year after the index date had higher predicted probabilities of high HCU compared to those without. The internal validation procedure revealed low optimism, indicating that the model was not overfit to the development sample. The prediction model showed good prediction performance and calibration (Table 3), with the calibration plot demonstrating that the predicted probabilities closely follow the observed probabilities across all deciles of predicted risk (Fig. 4A). After external validation, the prediction model continued to show good predictive performance (Table 3). Calibration was good, with the calibration slope indicating slightly conservative predicted risks and the calibration-in-the-large (CITL) indicating slight underestimation of risk on average (Table 3). The calibration plot shows that the predicted probabilities follow the observed probabilities for most deciles, with slight underestimation in the upper 3 deciles (Fig. 4B).

**Table 3:** Performance statistics of development, internal, and external validation of prediction models for patients with spinal disorders referred for secondary care evaluation.

Aspect	Measure	Development	Internal validation	External validation
Overall performance	*R* ^2^	0.27	0.26 (0.25, 0.27)	0.31
Discrimination	AUC	0.78	0.78 (0.77, 0.78)	0.81 (0.80, 0.81)
Calibration	CITL	0.00	−0.02 (−0.06, 0.02)	0.16 (0.12, 0.19)
	Slope	1.00	0.98 (0.95, 0.99)	1.08 (1.06, 1.11)

Development and internal validation cohort Norway (n = 9092, 44 imputed data sets). External validation cohort Denmark (n = 34,853, 44 imputed data sets). Parentheses are 95% confidence intervals.

AUC, area under receiver operating characteristics curve; CITL, calibration-in-the-large; *R*^2^, Nagelkerke pseudo-*R*^2^.

### 3.3. Additional analyses

Removing HCU previous year from the original model yielded an optimism-adjusted Nagelkerke *R*^2^-value of 0.11 (95% CI 0.10–0.13) and AUC of 0.69 (95% CI 0.67–0.70) for internal validation, and *R*^2^-value of 0.18 and AUC of 0.73 (95% CI 0.72–0.73) for external validation. The new model included all original predictors plus pain duration, spinal surgery history, and sick leave history, resulting in a Nagelkerke *R*^2^-value of 0.12 (95% CI 0.10–0.13) and AUC of 0.69 (95% CI 0.68–0.70) for internal validation, and *R*^2^-value of 0.17 and AUC of 0.72 (95% CI 0.72–0.73) for external validation. Performance statistics and calibration plots are in Supplementary Table S4 and Figure S3, http://links.lww.com/PR9/A367, coefficients for all models in Table S5, http://links.lww.com/PR9/A367, and prediction calculations in Box S1, http://links.lww.com/PR9/A367. Sensitivity analyses using CCA and the 90th percentile cutoff yielded results consistent with the main analyses (Supplementary Tables S5-S6 and Figure S4, http://links.lww.com/PR9/A367).

## 4. Discussion

In this study involving 9092 Norwegian and 34,853 Danish patients with spinal disorders, a prediction model for high all-cause HCU was developed and validated for use in secondary care settings. The model performed well upon external validation, with excellent discrimination and good calibration performance, affirming robustness across different health care contexts. The final model included 13 predictors covering all 3 domains of the Andersen's Behavioral Model of Health Services Use, predisposing, enabling, and need factors, highlighting the multidimensional nature of health care utilization.

### 4.1. Comparison with previous studies

To the best of our knowledge, this study is the first to develop, and internally and externally validate a prediction model specifically for this patient population in secondary care within a single study. Although prediction models for future HCU exist across various populations, most spinal disorder models focus on patient-reported outcome measures, often in surgical populations, providing valuable insights into recovery outcomes.^7,24,26,72^ Few studies, however, target patients managed conservatively, particularly in secondary care settings. For example, Chen et al.^11^ developed prediction models for spine-related primary care seeking and frequent health care visits among 15,000 individuals with self-reported chronic neck and low back pain in Norway in a general population setting, with C-statistics lower than ours (0.66–0.76), and no external validation. Similarly, George et al.,^25^ investigated nearly 600,000 U.S. individuals seeking physical therapy in primary care for a broader range of musculoskeletal pain, developing models based on 3 definitions of recurrent care, all showing good calibration (calibration slopes 1.014–1.061) but weak C-statistics (0.59–0.64). Whereas other studies used different predictors that do not fully encompass all 3 domains of Andersen's Behavioral Model of Health Services Use, our approach ensured the inclusion of predisposing, enabling, and need factors to enhance robustness and applicability.^3^

### 4.2. Interpretation and relevance

The external validation results indicate improved model discrimination compared to internal validation, suggesting strong predictive ability across 2 different populations from different countries with similar health care systems. Although calibration slightly decreased in external validation, the model's overall predictive capacity remained strong, suggesting relevance of predictors across populations and no overfitting. Although the model remains well-calibrated and generalizes effectively, minor recalibration, like adjusting the intercept for CITL, could enhance predictive accuracy in new settings, populations, and contexts. However, the improved discrimination in external validation supports the model's potential clinical utility, emphasizing its generalizability across populations. Although both datasets originated from secondary care settings and included patients with spinal disorders, differences in health care system structures, patient demographics, and HCU patterns between Norway and Denmark could influence the model's performance.^15^ Continued validation across diverse settings, populations, and contexts is recommended to thoroughly evaluate the model's transportability and effectiveness. Thus, our study may have direct clinical implications in secondary care settings, enabling calculation of individualized predicted probabilities. For example, the final model predicts that a woman with high values on all predictors has almost 10 times higher probability of high HCU during the follow-up year compared to 1 with low to moderate values (68.4% vs 7.0%) (Box 1).

We found that HCU in the previous year was the strongest predictor of high HCU 1 year later. Although our study utilized registry data to assess prior HCU, such data may not always be accessible in clinical settings. An alternative approach could involve asking patients about prior HCU, although this method may introduce inaccuracies due to recall bias and subjective reporting. To address this limitation, we developed and validated models excluding prior HCU as a predictor to ensure robustness and applicability in settings without comprehensive health care data. Area under receiver operating characteristics curves for internal validation were slightly below the threshold for acceptable discrimination, whereas those for external validation exceeded it. Although discrimination and overall performance were reduced compared to the original model, the alternative models still demonstrated some ability to differentiate between risk groups and predict future HCU.^61^ Sensitivity analyses using the 90th percentile and CCA were largely consistent with the main analyses, despite some differences in calibration and R^2^. This supports the robustness and validity of our model across thresholds.

### 4.3. Strengths and limitations

The main strength of this study was the development and validation of the prediction model using 2 large independent samples from 2 different countries with similar health care systems. We included a broad spectrum of candidate predictors and aligned them across both datasets, ensuring a high level of consistency and likely improving performance upon external validation compared to the development and internal validation. The main limitation of the study is the unavailability of contextual predictors of HCU, such as health care access, community demographics, including local variations in health care policy and financing, and the health beliefs and health behaviors of peers.^3^ Another limitation may be that counting consultations may not fully capture HCU variability. Although this provides a straightforward measure of service use, it does not account for the complexity and resource intensity of different consultation types. Using cost as a proxy offers a more nuanced view^16,28^ but may disproportionately emphasize high-cost services over frequent low-cost services, potentially skewing the understanding of health care demands. There is also a potential underestimation of HCU since private health care providers without reimbursement contracts are not registered in public registers, and data on radiologic consultations are lacking. Last, data collected during the COVID-19 pandemic may have influenced HCU patterns, potentially affecting both predictors and outcomes. This should be considered when interpreting the findings.

Overall, the model's strong external validation performance supports its utility in predicting high HCU, enhancing its applicability for health care planners and providing a reliable tool for discussing future care pathways.

### 4.4. Implications

The predictive model will help clinicians discuss health care use with patients. Clinicians can, for example, guide patients toward supported self-management strategies, which effectively reduce chronic spinal pain and associated disability,^6,17,73^ empowering patients to take control of their health and reducing reliance on health care services. High HCU can be beneficial when it reflects necessary care for complex health needs or leads to early intervention and better outcomes; however, it can also indicate unnecessary service use, or fragmented care without improved health outcomes.^8^ For example, previous studies have found that patients with spinal disorders referred to specialist evaluation in secondary care often continue using health care services for several years after the index date.^40,60^ This ongoing utilization may reflect persistent health care needs that do not necessarily result in recovery, as demonstrated in a previous study we conducted with patients from this population, where only 20% reported recovery after 6 months.^39^ Our model can also provide valuable insights for health care decision makers at an organizational level. Although direct application by health care service planners is limited, insights into predictors of HCU, such as nationality, education, and prior utilization, can guide resource allocation, inform policy development, and identify areas for interventions to optimize utilization. To ensure the model's applicability and relevance in clinical practice, it is essential to evaluate the model within implementation settings. The models excluding previous HCU as a predictor are more applicable in clinical settings where historical data cannot be obtained, although it comes at the cost of reduced predictive performance. If clinicians rely on self-reports for previous HCU, it is essential to establish clear and accurate categories to ensure reliable data collection.

## 5. Conclusion

We developed and validated a prediction model for high all-cause HCU among patients with spinal disorders visiting secondary care in Norway and Denmark. The model demonstrated acceptable discriminating ability upon internal validation and excellent discriminating ability upon external validation, showing promise as a tool for identifying patients at risk of high HCU.

## Disclosures

The authors have no conflict of interest to declare.

## Supplemental digital content

Supplemental digital content associated with this article can be found online at http://links.lww.com/PR9/A367.

## Supplementary Material
