# Supplementary material for: Predicting high health care use in patients with spinal disorder in secondary care: model development and validation
Source: Pain Rep. 2026 Jan 16;11(1):e1382. doi: 10.1097/PR9.0000000000001382 (PMC12815531; doi:10.1097/PR9.0000000000001382)
Supplement: SUPPLEMENTARY MATERIAL [file painreports-11-e1382-s001.pdf]

Supplemental digital content

List of Content:

Table S1: Definition of candidate predictors …………………………………. 1
Table S2: Definition of the comorbidity variable …………………………….. 5
Figure S1: Trace plots ……………………………………………………… 7
Figure S2: Density plots …………………………………………………… 8
Table S3: HCU before and after index consultation …………………………. 10
Table S4: Performance statistics additional models ..………………………... 10
Figure S3: Calibration plots for external validation in additional analyses ….. 11
Table S5: Prediction models for high HCU with OA coefficients …………… 11
Table S6: Performance statistics of sensitivity analysis models ……………... 13
Figure S4: Calibration plot for the 90th percentile outcome ………..……... 13
Box 1: Demonstration of prediction calculation, additional model 2 ………... 14

Supplementary Table S1 Definition of candidate predictors. List of the 22 candidate predictors used for model development, their measurement level and number of predictor parameters in the first modelling step. Categorised according to Andersen's Behavioral Model of Health Services Use [1]

Predictors	Definition and/or categories in development data	Data source development data	Definition and/or categories in validation data	Data source validation data	Properties in the model	Predictor parameters
Predisposing factors						
**Age**	-	H	-	R	Continuous	1
**Sex**	-	H	-	R	Factor:<br>Male = 0, female = 1	1
**Country of origin**	Norway (Native)<br>Europe<br>Africa<br>Asia<br>North and Central America<br>South America<br>Other	S	Denmark (Native)<br>Europe<br>Africa<br>Asia<br>North and Central America<br>South America<br>Other	R	Factor:<br>Native = 0, non-native = 1	1

**Marital status**	Married/registered partner<br>Cohabiting<br>Single	S	Cohabiting<br>Single	R	Factor:<br>Married/partner/cohabiting = 0, Single = 1	1
**Education**	*"What is your highest completed education?"*<br>Primary school (year 0-10)<br>Vocational high school (year 11-12/13)<br>Academic high school (year 11-13)<br>Higher education <4 years<br>Higher education ≥4 years	S	Highest completed education<br><br>Primary school<br>Vocational high school<br>Academic high school<br>Higher education <4 years<br>Higher education ≥4 years	R	Factor:<br>Lower secondary = 0, upper secondary = 1, university = 2	2
**Physical activity**	*"How is your activity level regarding training/movement/physical activity in your leisure time?"*<br>Sedentary<br>Light, for at least 4 hours per week<br>Moderate, for at least 4 hours per week<br>Hard, competitive several times per week	S	*"How physically active are you normally during your leisure time? What category fits best for you?"*<br>Sedentary<br>Light, for at least 4 hours per week<br>Moderate, for at least 3 hours per week<br>Hard, competitive several times per week	S	Factor:<br>Hard = 0, moderate = 1, light = 2, sedentary = 3	3
**Smoking daily**	*"Do you smoke daily?"*<br>Yes<br>No	S	*"Are you a smoker?"*<br>No<br>Only occasionally<br>Yes 1-4 cigarettes daily<br>Yes 5-14 cigarettes daily<br>Yes 15-24 cigarettes daily<br>Yes 25+ cigarettes daily	S	Factor:<br>No/only occasionally=0, Yes = 1	1
**Healthcare utilization previous year**	All face-to-face or video healthcare contacts in primary and secondary care. Simple communication, prescriptions, and administrative registrations are excluded.	R	All face-to-face or video healthcare contacts in primary and secondary care. Simple communication, prescriptions, and administrative registrations are excluded.	R	Factor:<br>Quartile 1 = 0, quartile 2 = 1, quartile 3 = 2, quartile 4 = 3	3
Enabling factors						
**Physically heavy work**	*"How physically demanding would you rate your work?"*<br>0-10 (Higher score equals more physically demanding work)	S	*"How physically demanding do you think your work is/was?"*<br>0-10 (Higher score equals more physically demanding work)	S	Continuous	1
**Work satisfaction**	*"How satisfied are you with your work?"*<br>0-10 score, higher score indicates higher satisfaction	S	*"If you assess your work tasks, management, salary, development opportunities and work colleagues as a whole, how satisfied are you with your work?"*<br>0-10, higher score indicates higher satisfaction	S	Continuous	1
**Applied for disability pension *or* claim issue**	*"Have you applied for disability pension?"*<br>Yes, no	S	*"Have you applied for disability pension because of pain in lower back, leg, neck, or upper back?"*	S	Factor: no = 0, yes (on either) = 1	1

	"Have you registered an insurance claim issue?"<br>Yes, no		Yes, no<br>*"Is there an insurance or other claim issue because of your pain?"*<br>Yes, no			
**Work status**	*"Current work status"*<br>Working<br>Student<br>Stay at home<br>Unemployed<br>On sick leave<br>Disability pension<br>Work assessment allowance<br>Retirement pension	S	*"What is your current work status?"*<br>Employed work full time or part time<br>"Flexjob" (work with special health considerations, similar to work assessment allowance)<br>Unemployed<br>Student<br>Job training (similar to work assessment allowance)<br>Early retirement pension<br>"Efterløn" (identical to retirement pension)<br>Retirement pension<br>Stay at home<br>Other<br>On sick leave:<br>*"Are you currently on sick leave because of pain in your back/neck/upper arm or leg?"*<br>Yes<br>No	S<br><br><br><br><br><br><br><br><br><br><br><br><br><br><br>S	Factor:<br>Working/student = 0, not in work force (stay at home/retirement pension/other) = 1, temporarily out of work force (current sick leave/unemployed) = 2, disability pension/work assessment allowance = 3	3
**Previous sick leave for similar complaint**	*"Have you been on sick leave for the same or similar complaint during the last two years?"*<br>No<br>Yes, once<br>Yes, 2-5 times<br>Yes, 6-10 times<br>Yes, more than 10 times	S	*"Have you been on sick leave because of pain during the last three months?"*<br>No<br>Yes	S	Factor:<br>no = 0, yes = 1	1
Need factors						
**Pain duration**	*Duration of current pain:*<br>No pain<br>Less than three months<br>Three to twelve months<br>One to two years<br>More than two years	S	*"Approximately when did your current pain start?"*<br>Date. Recoded to duration categories corresponding to the development study.	S	Factor:<br>no pain or less than three months = 0, three to twelve months = 1, more than twelve months = 2	2
**Pain intensity**	*"How would you rate your pain during rest the last week?"*<br>Numeric Rating Scale 0-10, higher score indicates more pain	S	*"How would you rate your typical pain during the last 14 days?"*<br>*or** *"How would you rate your typical leg pain (sciatica) during the last 14 days?"*	S	Continuous	1

			Numeric Rating Scale 0-10, higher score indicates more pain.			
**Disability**	Oswestry Disability Index (ODI) score or Neck Disability Index (NDI) score, depending on pain location. 0-100, higher score indicates more disability	S	Oswestry Disability Index score or Neck Disability Index score, depending on pain location. 0-100, higher score indicates more disability	S	Continuous	1
**Previous surgery**	Neck surgery, back surgery and/or pelvic surgery:<br>Yes<br>No<br>Don't know	S	Based on registry surgery codes for neck surgery, back surgery and / or pelvic surgery	R	Factor:<br>no = 0, yes = 1<br>"Don't know" are counted as missing	1
**Prescription pain medication**	*"How often have you used the following medication during the last 4 weeks?"*<br>Pain medicine with prescription: Not used, less than once per week, weekly but not daily, daily	S	Registered prescription during the last 4 weeks before index for the following medication groups:<br>Paracetamol, opioids, gabapentins, SNRIs, TCAs, muscle relaxants	R	Factor:<br>no = 0, yes = 1,	2
**Pain location**	Back pain (with and without radiculopathy)<br>Neck pain (with and without radiculopathy)<br>Both	H	Low back pain (with and without radiculopathy)<br>Neck pain, including thoracic pain (with and without radiculopathy)	S	Factor:<br>Low back pain = 0, neck pain = 1, both = 2	2
**Kinesiophobia / fear avoidance behaviour**	Fear Avoidance Beliefs Questionnaire-physical activity subscale, item 3: *"I should not do physical activities that (might) hurt my back"*<br>Answered at 0-6 scale, higher score indicates higher agreement. Transformed to a 0-10 scale to accommodate external validation scale**.	S	*"I should not do physical activities that (might) make my pain worse"*<br>0-10 score, 10 indicates higher agreement	S	Continuous 0-10	1
**Health-related quality of life**	EQ5D-3L (-0.594 to 1, higher score indicates higher health-related quality of life). From late 2020, the 5L version was implemented, reverse crosswalk values were computed.	S	EQ5D-5L (-0.594 to 1, higher score indicates higher health-related quality of life)	S	Continuous	1
**Comorbidity**	Comorbidities identified using Danish Multimorbidity Index and corresponding primary care (KUHR) diagnostic codes during the previous year.	R	Comorbidities identified using Danish Multimorbidity Index (secondary care diagnostic codes and prescription registry)	R	Continuous count of comorbidities	1
Maximum number of predictor parameters:						32

H, Healthcare provider; R, Registry; S, Self-reported.
* Some patients may not have much back pain, but the sciatic leg pain is more prominent and may for some be a more representative measure. If both questions were answered, we used the highest score
** We chose Item 3 from the Fear Avoidance Beliefs Questionnaire – Physical activity subscale for kinesiophobia because this was available in both samples. In the development sample. This single item has previously shown adequate criterion validity [5].

**Supplementary Table S2** Definition of the comorbidity variable. List of disease groups and their corresponding diagnostic codes for the development and internal validation sample, and external validation sample. The list and the corresponding ICD-10 and ATC codes are from the Danish Multimorbidity Index [2]. Most items overlap with the ICPC morbidity index list and corresponding ICPC-2 codes [3]. Those that do not overlap are extracted from the ICPC-2 diagnostic code database available from https://finnkode.helsedirektoratet.no/icpc2/search.						
		**Development and internal validation**	**External validation**			
**Category**	**Disease group**	**ICPC-2 diagnostic codes*^**	**ICD-10 diagnostic codes**	**Diagnosis time frame**	**Drug codes (ATC)**	**Prescription time frame**
**Circulatory**	Hypertension	K86, K87	I10-I13, I15	Ever	C02, C04, C07, C08, C09, C03	Twice last year
	Dyslipidemia	T93	E78	Last two years	C10	Twice last year
	Ischemic heart disease	K74, K76	I20-25	Ever	C01DA	Twice last year
	Atrial fibrillation	K78	I48	Ever		
	Heart failure	K77	I50	Ever		
	Peripheral artery occlusive disease	K92	I70-74	Ever		
	Stroke	K89, K90	I60-64, I69	Ever		
**Endocrine system**	Diabetes mellitus	T89, T90	E10-E14	Ever	A10A, A10B	Twice last year
	Thyroid disorder	T85, T86	E00-E05, E061-E069, E07	Last two years	H03	Twice last year
	Gout	T92	E79, M10			
**Pulmonary system and allergy**	Chronic pulmonary disease	R95, R96			R03	Twice last year
	Allergy	A92, D99, F71, R97, S88,			R06AX, R06AE07, R06AE09, R01AC, R01AD	Twice last year
**Gastrointensinal system**	Ulcer/chronic gastritis	D86, D87,	K221, K25-K28, K293-K295	Ever		
	Chronic liver disease	D97	B16-B19, K70-K74, K766, I85	Ever		
	Inflammatory bowel disease	D94	K50-51	Ever		
	Diverticular disease of intestine	D92	K57	Ever		
**Urogenital system**	Chronic kidney disease	U88	N03, N11, N18, N19	Ever		
	Prostate disorders	Y85	N40	Ever	C02CA, G04C	Twice last year
**Musculoskeletal system**	Connective tissue disorders	L99, S99, U88, R99	M05-M06, M08-M09, M30-M36, D86	Ever		
	Osteoporosis	L95	M80-M82	Ever	M05B, G03XC01, H05AA	Twice last year
	Painful condition	L18, L83, L84, L85, L86, L89, L90, L91, L92, N90, N95			N02BA51, N02BE, M01A, M02A	Four times last year

**Hematological system**	HIV/AIDS	B90	B20-B24	Ever		
	Anemias	B78, B80, B81, B82	D50-D53, D55-D59, D60-D61, D63-D64	Last two years		
**Cancers**	Cancer	A79, B72, B73, B74, D74, D75, D76, D77, L71, N74, R84, R85, T71, U75, U76, U77, W72, X75, X76, X77, Y77, Y78	C00-C43, C45-C97	Last five years		
**Neurological system**	Vision problem	F93, F94	H40, H25, H54	Ever		
	Hearing problem	H83, H84, H85, H86	H90-H91, H931	Ever		
	Migraine	N89	G43	Last two years	N02C	Twice last year
	Epilepsy	N88	G40-G47	Ever	N03	Twice last year
	Parkinson's disease	N87	G20-G22	Ever		
	Multiple sclerosis	N86	G35	Ever		
	Neuropathies	N94	G50-G64	Last two years		
**Mental health conditions**	Mood, stress-related, or anxiety disorders	P74, P75, P82	F32-F34, F40-F48	Last two years		
	Psychological distress	P76			N06A	Twice last year
	Alcohol problems	P15	F101-F109	Last two years		
	Substance abuse	P18, P19	F11-F16, F18-F19	Last two years		
	Anorexia/bulimia	P86	F50	Last two years		
	Bipolar affective disorder	P73	F30-F31	Ever	N05AN	Twice last year
	Schizophrenia or schizoaffective disorder	P72, P98	F20, F25	Ever		
	Dementia	P70	F00-F03, F051, G30	Ever	N06D	Twice last year

ICPC-2: International Classification of Primary Care-second version, ICD-10: International Classification of Diseases-version 10, ATC: Anatomical Therapeutic Chemical Classification System, HIV: human immunodeficiency virus, AIDS: Acquired immunodeficiency syndrome.
*Time frame for ICPC-2 diagnostic codes in primary care is one year before index consultation in secondary care.
^Includes subchapters. For example, chapter K86 also includes K86.0000, K86.0001, K86.0002.
To identify comorbidity, we used the ICPC-2 diagnostic codes in the development and internal validation sample, whereas we used ICD-10 diagnostic codes and ATC in the external validation sample. The diagnostic codes are based on the Danish Multimorbidity Index (DMI) [2]. For comorbidities with both diagnostic codes and drug codes, that patient did not have to fulfill both criteria to be identified as having the comorbidity.

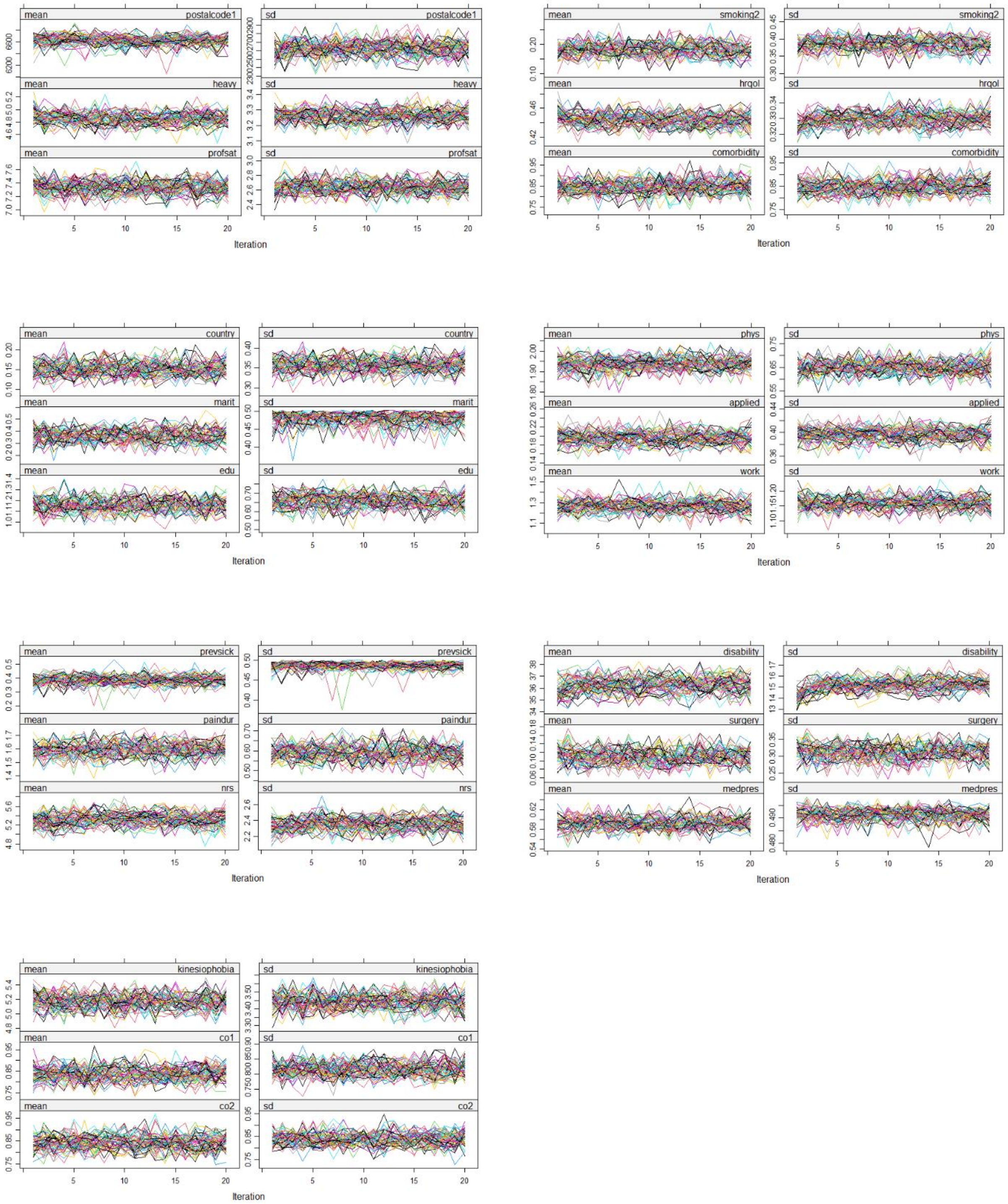

Supplementary Figure S1 Trace plots for checking convergence in the multiple imputation procedure for predictors and auxiliary variables.

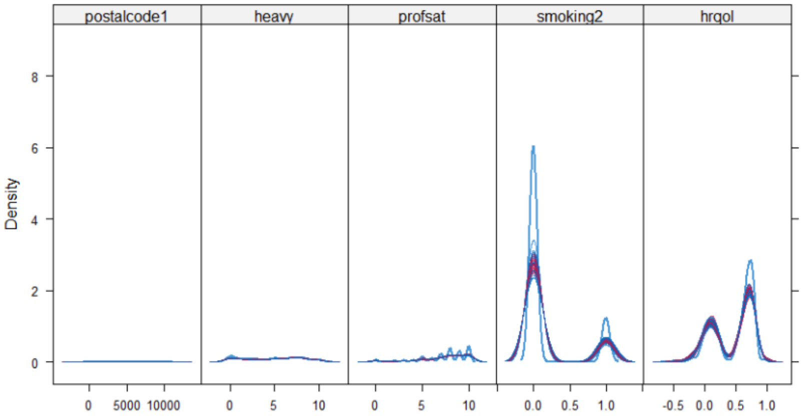
postalcode1
heavy
profsat
smoking2
hrqol
Density
0 2 4 6 8
0 5000 10000
0 5 10
0 5 10
0.0 0.5 1.0
-0.5 0.0 0.5 1.0

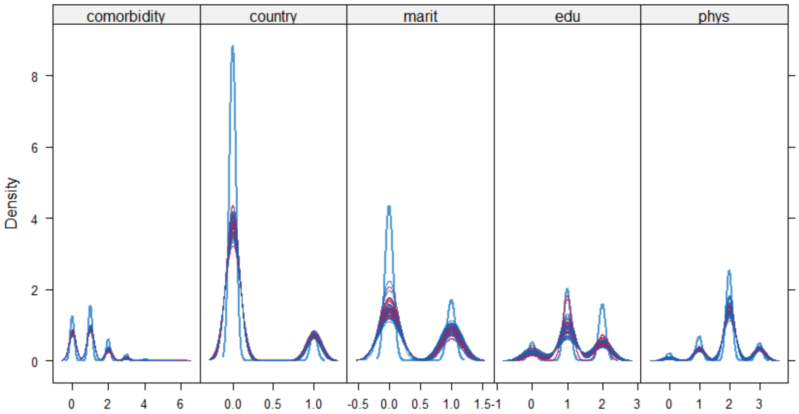
comorbidity
country
marit
edu
phys
Density
0 2 4 6 8
0 2 4 6
0.0 0.5 1.0
-0.5 0.0 0.5 1.0 1.5
-1 0 1 2 3
0 1 2 3

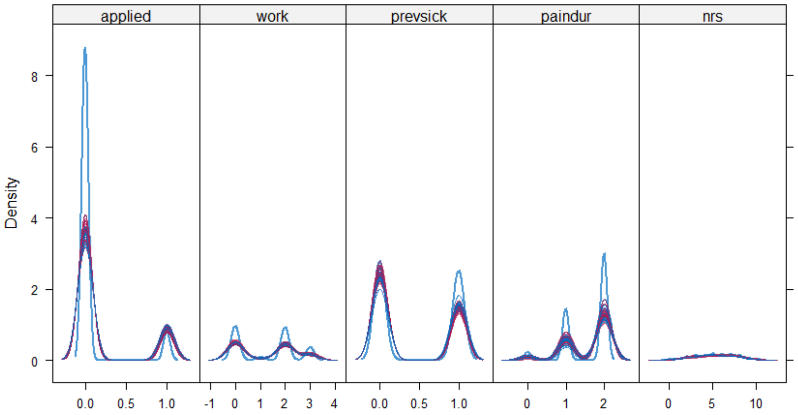
applied
work
prevsick
paindur
nrs
Density
0 2 4 6 8
0.0 0.5 1.0
-1 0 1 2 3 4
0.0 0.5 1.0
0 1 2
0 5 10

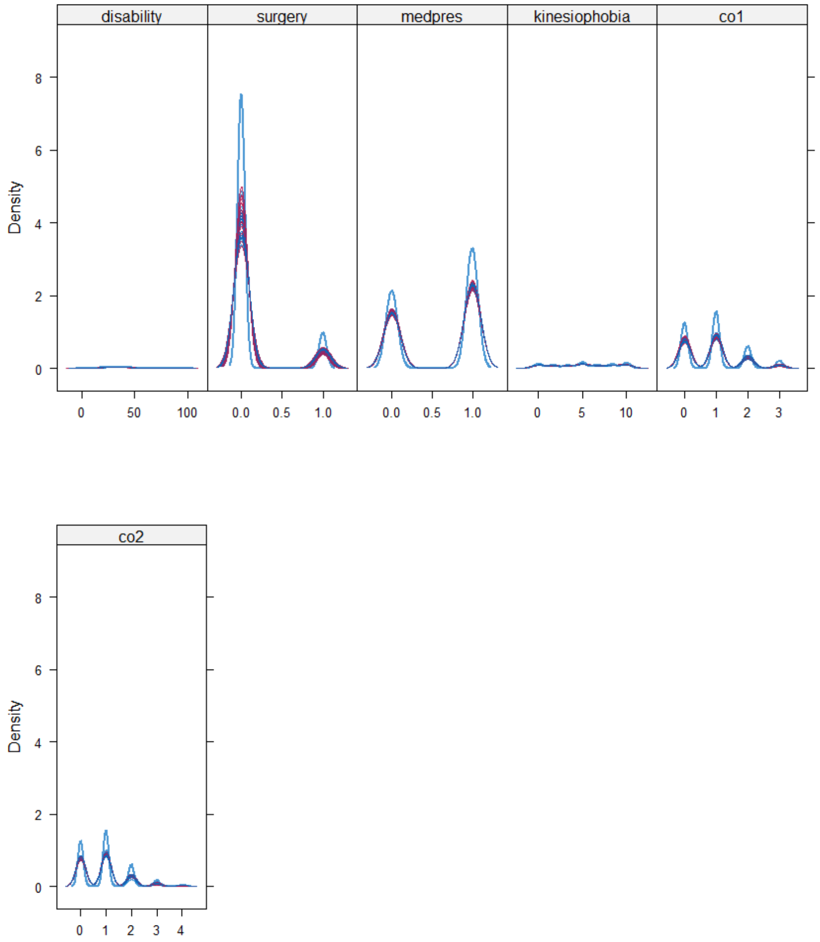

Supplementary Figure S2 Density plots of observed (blue) and imputed (red) values for predictors.

**Supplementary Table S3** HCU before and after index consultation in secondary care				
	**Development cohort**		**External validation cohort**	
	12 months before index	12 months after index	12 months before index	12 months after index
Primary care				
Total, median (IQR)	13 (6-23)	15 (7-26)	19 (11-29)	18 (10-30)
Total, mean (SD)	17.7 (17.0)	19.7 (18.9)	23 (19)	23 (20)
No visits, n (%)	103 (1.1)	234 (2.6)	0 (0)	313 (0,01)
GP, median (IQR)	5 (2-9)	5 (2-9)	8 (5-13)	7 (4-13)
GP, mean (SD)	6.8 (5.1)	6.1 (5.6)	10 (8)	9 (8)
PT, median (IQR)	0 (0-5)	0 (0-4)	0 (0-4)	0 (0-1)
PT, mean (SD)	5.6 (12.5)	5.6 (13.1)	4 (10)	4 (10)
Chiropractor, median (IQR)	0 (0-1)	0 (0-0)	0 (0-4)	0 (0-0)
Chiropractor, mean (SD)	2 (4.8)	0.9 (3.4)	3 (6)	1 (4)
Secondary care				
Inpatient and outpatient visits, median (IQR)	1 (0-4)	4 (1-10)	3 (1-8)	6 (2-12)
Inpatient and outpatient visits, mean (SD)	3 (6)	7 (9)	6 (9)	9 (11)

GP, General Practitioner; HCU, Healthcare Utilization; IQR, Interquartile Range; PT, Physical Therapy; SD, Standard Deviation.

**Supplementary Table S4** Performance statistics of internal and external validation of prediction models for patients with spinal disorders referred for secondary care evaluation **without the predictor HCU previous year**					
		**Additional model 1**		**Additional model 2**	
**Aspect**	**Measure**	**Internal validation**	**External validation**	**Internal validation**	**External validation**
Overall performance	R^2	0.11 (0.10, 0.13)	0.18	0.12 (0.10, 0.13)	0.17
Discrimination	AUC	0.69 (0.67, 0.70)	0.73 (0.72, 0.73)	0.69 (0.68, 0.70)	0.72 (0.72, 0.73)
Calibration	CITL	-0.03 (-0.11, 0.03)	0.11 (0.07, 0.16)	-0.04 (-0.11, 0.03)	0.10 (0.05, 0.14)
	Slope	0.96 (0.90, 1.01)	1.08 (1.05, 1.12)	0.96 (0.90, 1.01)	1.05 (1.01, 1.08)

AUC, Area under Receiver Operating Characteristics-curve; CITL, Calibration-in-the-large; HCU, Healthcare Utilization; R^2; Nagelkerke's pseudo-R^2.
Development and internal validation cohort Norway (n=9092, 44 imputed data sets). External validation cohort Denmark (n=34853, 44 imputed data sets Model 1 and 65 imputed data sets Model 2). Parentheses are 95% confidence intervals.
Additional model 1: Based on the originally developed model, excluding HCU previous year.
Additional model 2: Developed a new model without incorporating HCU previous year from the outset.

A) B)

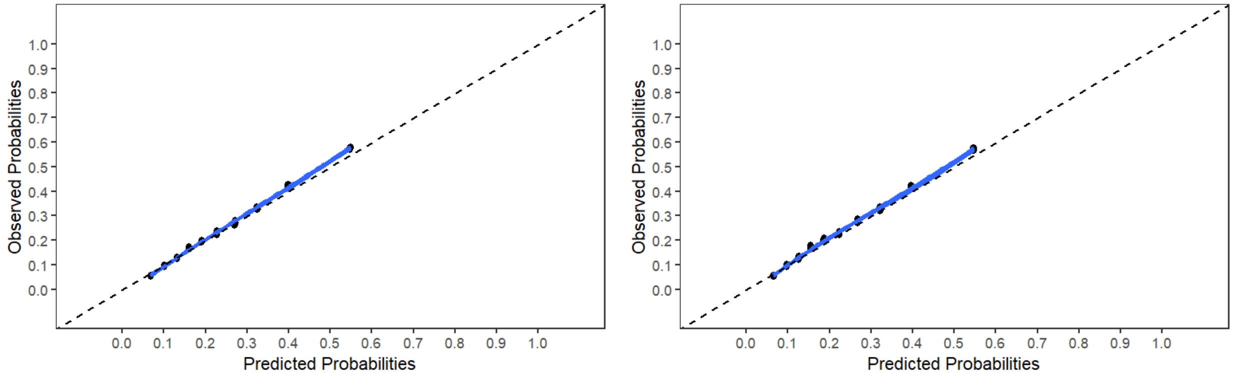

Supplementary Figure S3 Calibration plots for the external validation sample in additional analyses; A) Model 1 (the original model except the predictor HCU previous year) and B) Model 2 (development of a new model without incorporating HCU previous year).

Supplementary Table S5 Prediction models for high HCU with optimism adjusted (OA) coefficients

	Original model	Additional model 1	Additional model 2	CCA*	90th percentile†
Intercept	-3.29	-2.57	-2.85	-3.20	-4.66
Predisposing factors					
Sex female (ref: male)	0.47	0.59	0.59	0.45	0.41
Non-native origin (ref: native)	-0.19	-0.21	-0.20	-0.26	-
Education level (ref: primary school)					
High school	0.11	0.18	0.18	-0.08	-
Higher education	0.30	0.38	0.38	0.08	-
Physical activity level (ref: hard)					
Moderate	-0.03	-0.07	-0.08	-0.01	-
Light	0.08	0.05	0.02	0.07	-
Sedentary	0.29	0.19	0.17	0.37	-
Smoking (ref: non-smoking)	-0.14	-0.26	-0.25	-0.14	-0.26
HCU previous year (ref: Q1)					
Q2	0.62	-	-	0.62	0.68
Q3	1.16	-	-	1.16	1.50
Q4	2.33	-	-	2.33	2.90
Work satisfaction (range 0-10)	-	-	-	-0.03	-

Enabling factors					
Working status (ref: working/student)					
Stay at home/retired	0.46	0.63	0.67	0.55	0.29
Sick leave/unemployed	0.46	0.66	0.64	0.55	0.34
Work assessment allowance/ disability pension	0.28	0.57	0.56	0.37	0.34
Previous sick leave (ref: no previous sick leave)	-	-	0.17	-	-
Need factors					
Pain duration (ref: <3 months)					
3-11 months	-	-	0.26	-	-
≥12 months	-	-	0.25	-	-
Pain intensity (NRS range 0-10)				0.40	-
Disability (ODI/NDI range 0-100)	0.01	0.01	0.01	0.01	0.01
Previous surgery in neck, back or pelvis (ref: no previous surgery)	-	-	0.14	-	-
Taking prescription pain medication (ref: not taking prescription pain medication)	0.10	0.14	0.13	-	0.11
Pain location (ref: back pain)					
Neck pain	0.23	0.24	0.25	0.25	0.29
Back and neck pain	0.25	0.29	0.29	0.19	0.24
Kinesiophobia (range 0-10)	-0.02	-0.02	-0.02	-	-
HRQoL (EQ5D range -0.594 to 1)	-0.48	-0.49	-0.49	-0.42	-0.53
Comorbidity (range 0-6)	0.09	0.24	0.24	0.11	-

CCA, Complete Case Analyses; EQ5D, EuroQol-5 Dimension; HCU, Healthcare Utilization; HRQoL, Health-related quality of life; NDI, Neck Disability Index; NRS, Numeric Rating Scale; ODI, Oswestry Disability Index.
OA coefficients are adjusted after internal validation through 500 bootstrap samples for the original model and 100 bootstrap samples for the Additional model 1 and 2.
Additional model 1: Based on the originally developed model, excluding HCU previous year.
Additional model 2: Developed a new model without incorporating HCU previous year from the outset.
* CCA included 5091 patients (56% of the sample). Work satisfaction and pain intensity emerged as additional predictors, while taking pain medication and kinesiophobia were no longer included. The effect of higher education was reduced, and the effect of high school education reversed direction.
† A new model with 90th percentile cutoff was developed: nationality, education level, kinesiophobia, and comorbidity were no longer included, while higher HCU in the previous year showed a stronger effect.

**Supplementary Table S6** Performance statistics of CCA and 90th percentile outcome models				
**Aspect**	**Measure**	**CCA**	**90th percentile** (from original model)*	**90th percentile** (new model)
Overall performance	R^2	0.26	0.07	0.25
Discrimination	AUC	0.78	0.82	0.82
Calibration	Slope	-	-	1.0

AUC; Area under Receiver Operating Characteristics-curve; CCA, Complete Case Analyses; HCU, Healthcare Utilization; R^2, Nagelkerke's pseudo- R^2.
*The original model was tested using the original model developed for the 75th percentile on the 90th percentile outcome.

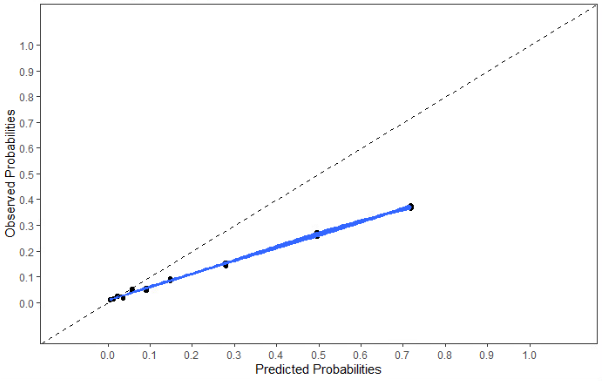

Supplementary Figure S4 Calibration plot for the outcome variable defined as the 90th percentile of high HCU, calculated including the original intercept, showing overestimation of risk in the upper three deciles.

Demonstration of equations for predicting high HCU within 12 months in individual patients with spinal disorders, based on the additional model without previous HCU

$$
\begin{aligned}
&High\ HCU \\
&= -2.85 + sex(0.59\ if\ female) + country\ of\ origin(-0.20\ if\ non-native) \\
&+ education\ level(0.18\ if\ high\ school, 0.38\ if\ higher\ education) \\
&+ physical\ activity\ level(-0.08\ if\ moderate, 0.02\ if\ light, 0.17\ if\ sedentary) \\
&+ smoking(-0.25\ if\ smoker) \\
&+ work\ status(0.67\ if\ stay\ at\ home, 0.64\ if\ sick\ leave, 0.56\ if\ work\ assessment\ allowance) \\
&+ previous\ sick\ leave(0.17\ if\ yes) + pain\ duration(0.26\ if\ 3-11\ months, 0.25\ if \\
&\geq 12\ months) + 0.01 * (disability) + previous\ spinal\ surgery(0.14\ if\ yes) + -0.49 * (HRQoL) \\
&+ prescription\ medicine\ use(0.13\ if\ yes) \\
&+ pain\ location(0.25\ if\ neck\ pain, 0.29\ if\ back\ \&\ neck\ pain) + -0.02 * (kinesiophobia) + 0.24 \\
&* (comorbidity)
\end{aligned}
$$

Example 1

Patient 1 is a Norwegian woman with primary school education, currently on sick leave. She is a smoker, lives a sedentary lifestyle, and suffers from back pain. She had previous sick leave for the same reason, previous spinal surgery, and pain duration for ≥12 months. She has a disability score of 56, $HRQoL$ score of 0.10, and kinesiophobia score of 7. She is using prescribed medication and has 2 comorbidities.

High HCU would be estimated as:

$$
\begin{aligned}
High\ HCU = &-2.85 + (0.59 * 1)(female) + (-0.20 * 0)(native) + 0(primary\ school\ education) \\
&+ 0.17(sedentary\ lifestyle) + (-0.25 * 1)(smoker) + 0.64(sick\ leave) \\
&+ 0.17(previous\ sick\ leave) + 0.25(pain\ duration) + (0.01 * 56)(disability) \\
&+ 0.14(previous\ spinal\ surgery) + (-0.48 * 0.10)(HRQoL) + (0.10 \\
&* 1)(prescription\ medicine\ use) + 0(back\ pain) + (-0.02 * 7)(kinesiophobia) \\
&+ (0.09 * 2)(comorbidities) = -0.488
\end{aligned}
$$

$$
\text{Probability of high HCU} = \frac{\exp(-0.488)}{1 + \exp(-0.488)} = 0.380
$$

Thus, patient 1 has a 38.0% probability of high HCU in the next 12 months

Example 2

Patient 2 is a Norwegian woman with higher education who is currently working. She is a non-smoker, engages in hard physical activity, and suffers from back pain. She had no previous sick leave for the same

reason, no previous spinal surgery, and pain duration for <3 months. She has a disability score of 35, $HRQoL$ score of 0.65 and a kinesiophobia score of 5. She is not using prescribed medication and has no comorbidities.

High HCU would be estimated as:

$$High\ HCU = -2.85 + (0.59 * 1)(female) + (-0.20 * 0)(native) + 0.38(higher\ education) + 0(hard\ physical\ activity) + (-0.25 * 0)(non - smoker) + 0(working) + (0.17 * 0)(previous\ sick\ leave) + 0(pain\ duration) + (0.01 * 35)(disability) + (0.14 * 0)(previous\ spinal\ surgery) + (-0.48 * 0.65)(HRQoL) + (0.10 * 0)(prescription\ medicine\ use) + 0(back\ pain) + (-0.02 * 5)(kinesiophobia) + (0.09 * 0)(comorbidities) = -1.942$$

$$\text{Probability of high HCU} = \frac{\exp(-1.942)}{1 + \exp(-1.942)} = 0.125$$

Thus, patient 2 has a 12.5% probability of high HCU in the next 12 months

Supplementary Box S1 Demonstration of prediction calculation based on addition model 2.
HCU, Healthcare Utilization; HRQoL, Health-Related Quality of Life

References

[1] Andersen RM. National Health Surveys and the Behavioral Model of Health Services Use. Med Care 2008;46(7):647-653.

[2] Prior A, Fenger-Grøn M, Larsen KK, Larsen FB, Robinson KM, Nielsen MG, Christensen KS, Mercer SW, Vestergaard M. The Association Between Perceived Stress and Mortality Among People With Multimorbidity: A Prospective Population-Based Cohort Study. Am J Epidemiol 2016;184(3):199-210.

[3] Sandvik H, Ruths S, Hunskaar S, Blinkenberg J, Hetlevik Ø. Construction and validation of a morbidity index based on the International Classification of Primary Care. Scand J Prim Health Care 2022;40(2):305-312.
